# Book Reviews

**Published:** 1855-12

**Authors:** 


### THE EPITOME OF SANITARY LITERATURE.

### CLIMATE, WEATHER, AND DISEASE.*

An attempt to show the connexions which exist between the variations of climate and weather, and the development of diseases, forms at all times an important inquiry when carefully and properly conducted. For as the human body presents many complex hydrostatic problems, as well as chemical ones, it is of necessity modified considerably by the variations of temperature, barometric pressure, and other external causes. In the spirit of a true and faithful inquirer, and with a store of classical learning rarely met with, Mr. Haviland has, in the work before us, brought together a vast amount of research, terse argument, and instructive information.

The following excellent ideas are offered in the opening chapter on climatology.

"In studying climate we study man; for in tracing its effects in all their variety on the human frame we make ourselves acquainted with his laws, customs, psychical and physical capabilities, vices, virtues, and all that appertains to that Protean animal. We find it in one region depressing, and in another elevating his various attributes: here it seems to endue his person with capacity for excessive delight, there it blunts his nerves and reduces psychical sensibility; in one region is it an element in the cause of slavery, in another does it invigorate man, and stimulate him to stand up for his own freedom, and to obtain redress for others.

"But to follow climate through all its innumerable effects on man, would be to attempt his history, to trace the progress of his mind, and to show how the arts, sciences, ethics, religion, war, and servitude, are regulated by it,—in fact, to write everything that we know about him."

Such thoughts might have emanated from Rousseau.

Mr. Haviland is a great admirer of Hippocrates, and few medical scholars are better read in this great master than the author

* Climate, Weather, and Disease: a Sketch of the Opinions of the most celebrated Ancient and Modern Writers with regard to the Influence of Climate in producing Disease. By Alfred Haviland. London: 1855. Churchill.

before us. Those who wish to have a clear and concise idea of the "Iatro-meteorology" of the old Esculapian, cannot do better than read the work under notice. At the same time, the book is not a simple history; it includes a vast amount of information of an original kind, and blends the present with the future in a manner at once philosophical, interesting, and instructive.

### PSYCHOLOGICAL MEDICINE.*

Dr. Noble is one of those mental philosophers who, after having adopted the views of Gall and Spurzheim, has, on more deliberate consideration, recanted in great part, and now believes that the doctrine of separate organs for particular faculties of the mind, can hardly, in the present state of knowledge, be regarded as scientific truth. Dr. Noble has enlarged and sound views on the physiology of the brain, and on mental disorders. He shows, that congenital idiotcy and epileptic imbecility occur amongst the poorer classes very largely from poor diet and defective hygiene generally. The twelfth chapter of Dr. Noble's book, on "The Moral Treatment of the Insane", is one of those instructive essays which so characterise the first class medical literature of the present period. Here is an admirable passage on the duties of the physician.

"I submit that medical men must not, in their practice, be regarded as leaving off treatment, or as abandoning a patient to nature, because blood-letting, counter-irritation, and drugs, may cease to be employed. It is but a low estimate to form of the medical character, to recognise in the physician or surgeon merely the apothecary or the cupper. It is the office of the medical adviser to guide and direct a patient at all times, and under all circumstances, in the matter of health and disease; pointing out to him when in health the way to continue so; and when sick, the course whereby health may be recovered. And whatever the process which the preservation of health or the removal of sickness may involve, it is the practitioner's business to direct. This may be simply a day or two's rest in bed, or it may be a tour for recreation: a course of purgatives may be indicated, or a temporary use of bitters or of iron; an extended or more diversified social intercourse may be the requirement, or a partial withdrawal from excitement of this description; it may be an engagement of the mind in some special pursuit encouraging hope, and exciting pleasing anticipation; or it may happen that a certain wild enthusiasm needs to be moderated. But whatever it be, if it influence health, it comes clearly within the province of the medical man. And so long as the physician engages himself in watching his patient for the patient's own good, so long as he maintains a directing control over his course of life, he cannot be justly said to have left his patient to nature, or to have abdicated his proper functions,

* Elements of Psychological Medicine. By Daniel Noble, M.D. Second Edition. London: 1855. Churchill.

although for weeks he should neither prescribe a drug, nor abstract a drop of blood, nor irritate a square inch of skin."

Truly so; and the sooner the world takes advice so logical, liberal, and consonant with common-sense as Dr. Noble's, the sooner will it learn what the science of medicine really means; the sooner will it appreciate the physician from the "curer" and the quacksalver, and the more will it advance in both moral and physical health. We recommend the work before us to all thoughtful and progressive readers. They will have their reward for their pains, or we had rather said for their pleasure, since the work is written in the most pleasant, classical, and masculine English.

### PERSONAL AND DOMESTIC HYGIENE.*

MR. BEALE has already written a useful popular work on the laws of health. In the present book he addresses himself principally to the working classes, and in a simple style lays before them much useful information on cleanliness, diet, the physical training of children, exercise. "Until the whole population," says our author, "can respect themselves by having not only decent clothing, but clean and healthy homes, and more than one room for a whole family,—until this can be done, I fear our religious and educational establishments are in vain, in regard to their influence on the largest class, and that which most needs them."

### RULES FOR EATING AND DRINKING †

DR. BUDD's work is intended mainly for the medical profession; but it contains a few rules in regard to eating and drinking, which it were well to make known to all classes of readers.

*Rules for late diners.*—"The substantial meals should be separated by an interval sufficient to allow the stomach to exert its power. Abstinence from food should never be so protracted as to induce a sense of exhaustion, for fasting weakens the digestive power. If then the interval between breakfast and dinner be long, a light luncheon should be taken; if the dinner be in the middle of the day, a light supper should be taken. The last heavy meal should be some hours before bed time."

*Alcoholic Liquors.*—Dr. Budd is no very zealous friend of teetotalism. On this point he says: "Spirits and the thin wines are best suited to gouty persons, or to men in the middle of life who are of gouty families; persons who would do well to abstain from malt liquors. Malt liquors, from containing more of the elements of nutrition, are especially suited to agricultural labourers and others, whose work is hard, and whose diet is scanty and not sufficiently varied. The liquor that is most generally wholesome is wine. Tea and coffee are not open to the same objections as fermented drinks; but they are doubtless commonly taken to a very injurious

---

* Personal and Domestic Hygiene: addressed especially to the Working Classes. By LIONEL J. BEALE. London: 1855. Churchill,

† On Organic Diseases and Functional Disorders of the Stomach. By GEORGE BUDD, M.D., F.R.S. London: 1855. Churchill.

excess, by nervous or excitable persons in the upper or lower classes, who do not counteract their influence by bodily labour."

### CHOLERA—WATER SUPPLY.*

THE Report on the Cholera Outbreak in St. James is composed of the labours of a committee, of whom the Rev. H. Whitehead, Dr. Lankester, Dr. Snow, Dr. King, and several other gentlemen were members. In carrying out their labours, the committee have proceeded as men really in earnest; and we ought to receive their remarks with great respect. The main point in the Report is, that after due deliberation and inquiry, they have agreed to the opinion that by far the most probable cause of the outbreak of cholera in St. James, was the drinking of impure water, derived from the Broad-street pump, of cholera notoriety. This, as most of our readers are aware, was Dr. Snow's original hypothesis.

The labours of the Rev. Mr. Whitehead in this extended inquiry are beyond all praise.

### CHOLERA IN RELATION TO METEOROLOGICAL CHANGES.†

MR. GLAISHER'S Report was made to the President of the Board of Health. It is an able document, and does infinite credit to the industry and the candour of the most distinguished of all British meteorologists.

We subjoin Mr. Glaisher's conclusions, as he gives them himself at the end of his laborious inquiry:

"In the year 1854 the pressure of the atmosphere was great; the temperature generally high; sky overcast; direction of the wind N.E. and S.W., and the velocity of the air was less by one-half than its average for some time before; and at the time of the greatest mortality from cholera, the barometer reading was remarkably high, and the temperature above its average; a thick atmosphere, though at times clear, everywhere prevailed; weak positive electricity; no rain. In low places a dense mist and stagnant air, with a temperature in excess; temperature of the Thames water high; a high night London temperature; a small daily range; an absence of ozone, and no electricity.

"The three epidemics of 1832-48 and 54 were attended with a particular state of atmosphere, characterized by a prevalent mist, thin in high places, dense in low. During the height of the epidemic, in all cases, the reading of the barometer was remarkably high, and the atmosphere thick. In 1849 and 1854, the temperature was above its average, and a total absence of rain, and a stillness of air amounting almost to calm, accompanied the progress of the disease on each occasion. In places near the river, the night temperatures were high, with small diurnal range, a dense torpid

* Report on the Cholera Outbreak in the Parish of St. James, Westminster, during the Autumn of 1854. Published for the Vestry. London: Churchill.

† Report on the Meteorology of London, and its Relation to the Epidemic of Cholera. By JAMES GLAISHER, F.R.S. Blue Book.

mist, and air charged with the many impurities arising from the exhalations of the river and adjoining marshes, a deficiency of electricity, and, as shown in 1854, a total absence of ozone, most probably destroyed by the decomposition of the organic matter with which the air in these situations is strongly charged.

"In 1849 and 1854, the first decline of the disease was marked by a decrease in the readings of the barometer, and in the temperature of air and water; the air, which previously for a long time had continued calm, was succeeded by a strong S.W. wind, which soon dissipated the former stagnant and poisonous atmosphere. In both periods at the end of September, the temperature of the Thames fell below 60°, but in 1854 the barometer again increased, the air became again stagnant, and the decline of the disease was considerably checked. It continued, however, gradually to subside, although the months of November and December were nearly as misty as that of September. By the close of the year diarrhœa and cholera had subsided, but a high rate of mortality still continued.

"The co-existence of cholera with coincident meteorological phenomena is, to say the least of it, remarkable; so is the stagnant atmosphere prevalent during the time of cholera in each of the three periods, and which would seem to be a necessary condition to the activity of the disease.

"The inimical nature of the influence it exercises upon the public health, I regard as intimately connected with the state of the water and the marshes, which in the preceding pages are shown to be large evaporating surfaces for every description of poisonous exhalations. Impure water and impure air are inseparable, for the impurities of the former will be concentrated into the surrounding atmosphere, and there remain, unless rapidly dispersed under favourable atmospheric conditions.

"The agency of the river in fostering diseases is confirmed by the history of cholera just traced, and which we find to have been most fatal in low situations, and in London in those places on the south side of the Thames which afford an undisturbed lodgment for the reception of the air charged with the poisonous elements from evaporation and exhalation. The effect of a gentle wind is to float this atmosphere to enclosed spots, where its malignity becomes concentrated."

### THE CHOLERA EPIDEMIC OF 1854.*

THE Appendix published by the Board of Health, contains the report of Mr. Glaisher, above referred to, and reports from Drs. Hassall and R. D. Thomson, Mr. Rainey, and other scientific observers.

These reports are in many respects more than ordinarily valuable. In speaking of the atmosphere of a cholera ward, Dr. Thomson sums up as follows.

---

* Appendix to the Report of the Committee for Scientific Inquiries in Relation to the Cholera Epidemic of 1854. Board of Health.

"From the results which have been obtained in the course of the present researches, the following deductions may be drawn: —

"1. That in the atmosphere of a cholera ward, mechanical matters were diffused throughout the air derived from the inmates, that sporules of fungi and germs of vibriones, or vibriones themselves were obtained by filtration from the atmosphere—all of these bodies being adulterations, so to speak, of the pure oxygen and nitrogen, which alone constitute the wholesome predominating constituents of the elastic fluids destined for respiration.

"2. That from a ward only partially filled with patients affected with cholera, substances were separated which were mechanically dispersed to the very summit of the apartment, mixed with fungi or their sporules, while no vibriones, unless in the form of faint traces, could be detected.

"3. That in the atmosphere of an empty ward, communicating however with a ward containing cholera patients, mechanical matters were obtained and traces of fungi, and perhaps of vibriones.

"4. That in the external air adjacent to an hospital, substances mechanically distributed were likewise found, and sporules with fungi were also detected to a considerable extent, but no vibriones.

"5. That in the atmosphere of a sewer, bodies were also found in mechanical diffusion associated with sporules, fungi, and vibriones.

"6. That the air contained under the three first conditions from wards possessed an acid reaction, that the external atmosphere likewise indicated a similar chemical condition, and that the sewer atmosphere was alone alkaline.

"7. That although animal and vegetable life seem unequivocally to be diffused through cholera atmospheres, it would be premature to infer a connexion between the disease and these organisms until comparative trials have been extensively made on other conditions of air; and that the present researches must be only considered as a single stone placed as a contribution towards the foundation of a larger structure."

In regard to the water supply, Dr. Hassall thus remarks.

"The conclusions to be deduced from the preceding Microscopical Examinations of different Pump and Well Waters, principally obtained from Cholera Districts, are the following: —

"That nearly all of the well waters examined were of a very impure description.

"That seven of them abounded in animal and vegetable productions belonging to different classes, including entomostraceæ, annelida, infusoriæ, diatomaceæ, and fungi, and they also contained dead organic matter of different kinds.

"That at least two of the waters were evidently contaminated with sewage matter, which had principally assumed the form of nitrates.

"That in none of the waters was any production met which could be supposed to exert any influence in giving rise to cholera, although in several of them vibriones were detected."

Dr. Hassall likewise conducted a microscopical inquiry into the pathology of cholera. On this point the result he arrived at was, that the only fluid of the cholera patient which was uniformly found to contain living organic productions was the rice water discharge, which even while enclosed in the intestines, swarmed with vibriones, the presence of which exhibits at least a proneness to rapid decomposition.

### INTRAMURAL INTERMENTS.*

It is well known that Archdeacon Hale has expressed certain strong views against the present system of extramural interment, and against the views of those who have exposed the graveyard nuisances of London. Dr. Letheby, the medical officer of health for the city of London, has canvassed the Archdeacon's arguments in a learned and masterly manner. Dr. Letheby repeats in a few words the facts which have often been narrated, that the decomposition of dead bodies gives rise to gaseous products, which, if inhaled, must prove deleterious to health.

We sympathise fully with all those sanitary reformers, of whom Mr. Walker was the first, who decry the semibarbarous act of half entombing heaps of dead in the homes of the living. But, as we have often pointed out, the system of extramural interments is in its turn becoming a serious social evil. The great question of burial must soon engage the attention of the legislature.

The state of the graveyard of St. Andrew's, Holborn, is revolting. The ground is above its natural level, the soil is saturated with decomposing organic matter, the coffins covered with two feet of earth, and but for some few planks put to the railings to prevent the fall of earth, the passers by on the public road, might be favoured with a shower of bones and unctuous soil. There is literally here only a plank between life and death.

We congratulate Dr. Letheby in having inaugurated his sanitary jurisdiction over the city with two such useful documents.

### A HOUSEHOLD WATER FILTER.†

The Report on the New River Water is very favourable. Dr. Letheby thus describes a simple household filter:—"A large zinc funnel, capable of holding a gallon of water, is placed over a vessel from which the filtered water can be easily drawn off. The stem of the funnel is packed to the depth of five inches with fine white sand, and above this is a stratum, two inches thick, of coarsely ground animal charcoal—such as sugar-bakers employ. The sand and charcoal are to be well washed before they are put into the funnel, and a disc of perforated zinc is to be placed at the bottom of the stem to prevent the sand from running out. An instrument made in this way will keep in action a long time; when it fails, it is easily repaired by rewashing the sand and charcoal."

---

* Reports of the Medical Officer of Health for the City of London, on the Injurious Effects of Intramural Burial, and on the State of the Churchyard of St. Andrew's, Holborn.

† Report of the Medical Officer of Health of the City of London on the New River Water.

